# Factors influencing the adoption of self-management solutions: an interpretive synthesis of the literature on stakeholder experiences

**DOI:** 10.1186/s13012-015-0350-x

**Published:** 2015-11-13

**Authors:** J. Harvey, S. Dopson, R. J. McManus, J. Powell

**Affiliations:** 1 Nuffield Department of Primary Care Health Sciences, University of Oxford, Radcliffe Observatory Quarter, Woodstock road, Oxford, OX2 6GG UK; 2 Saïd Business School, University of Oxford, Park End Street, Oxford, OX1 1HP UK

**Keywords:** Human values, Self-management, Chronic illness, Barriers, Facilitators, Stakeholder experiences, Sensemaking

## Abstract

**Background:**

In a research context, self-management solutions, which may range from simple book diaries to complex telehealth packages, designed to facilitate patients in managing their long-term conditions, have often shown cost-effectiveness, but their implementation in practice has frequently been challenging.

**Methods:**

We conducted an interpretive qualitative synthesis of relevant articles identified through systematic searches of bibliographic databases in July 2014. We searched PubMed (Medline/NLM), Web of Science, LISTA (EBSCO), CINAHL, Embase and PsycINFO. Coding and analysis was inductive, using the framework method to code and to categorise themes. We took a sensemaking approach to the interpretation of findings.

**Results:**

Fifty-eight articles were selected for synthesis. Results showed that during adoption, factors identified as facilitators by some were experienced as barriers by others, and facilitators could change to barriers for the same adopter, depending on how adopters rationalise the solutions within their context when making decisions about (retaining) adoption. Sometimes, when adopters saw and experienced benefits of a solution, they continued using the solution but changed their minds when they could no longer see the benefits. Thus, adopters placed a positive value on the solution if they could constructively rationalise it (which increased adoption) and attached a negative rationale (decreasing adoption) if the solution did not meet their expectations. Key factors that influenced the way adopters rationalised the solutions consisted of costs and the added value of the solution to them and moral, social, motivational and cultural factors.

**Conclusions:**

Considering ‘barriers’ and ‘facilitators’ for implementation may be too simplistic. Implementers could instead iteratively re-evaluate how potential facilitators and barriers are being experienced by adopters throughout the implementation process, to help adopters to retain constructive evaluations of the solution. Implementers need to pay attention to factors including (a) cost: how much resource will the intervention cost the patient or professional; (b) moral: to what extent will people adhere because they want to be ‘good’ patients and professionals; (c) social: the expectations of patients and professionals regarding the interactive support they will receive; (d) motivational: motivations to engage with the intervention and (e) cultural: how patients and professionals learn and integrate new skills into their daily routines, practices and cultures.

## Introduction

Self-management (SM) of chronic disease, where patients manage their illness independently but with the support of health care professionals, has been shown to be cost-effective in a range of conditions [1–3]. SM has therefore become the focus of national healthcare polices including the UK’s expert patient programme [4] and many care interventions [5–7]. Hence, there is a strong attention on the implementation and adoption of SM solutions, which can range from keeping a diary of activities using notebooks to a complex telehealth system. Key foundations of SM include (a) patients’ capability to engage with certain material innovations which may include documented protocols, guides and instructions, electronic devices and diaries; (b) patients’ effective engagement with healthcare professionals, peers and family for support and (c) clinicians’ effective promotion of SM practices to patients [8–10].

Whilst self-management solutions have shown benefit in academic evaluations, they have been challenging to implement. Self-management studies have sought to understand the reasons for implementation challenges from perspectives such as those of patients, professionals or health systems along with disease specific issues [11–14]. Comprehensive literature reviews and original studies on implementation in the wider healthcare literature have also sought to understand factors that contribute to successful implementation [15–19]. Many of these studies have drawn conclusions about the critical ‘barriers’ and ‘facilitators’ to implementation. Despite awareness of these, many solutions still fail in their uptake, adoption and diffusion [15, 20]. Clearly, the identification of such factors alone has not been sufficient. This study therefore focussed on reviewing and conducting an interpretive analysis of the literature regarding experiences of people adopting any self-management solution. In examining the contextual factors and specifically how stakeholders interpreted and evaluated the solutions, we applied a theoretical framework of ‘sensemaking’.

Sensemaking is an ongoing process of how people rationalise connections between themselves, events and places and is argued keeps cognition and action together [21–23]. Weick, Sutcliffe and Obstfeld [23] identify that sensemaking has seven characteristics, which include sensemaking organises flux; starts with bracketing; is guided by mental models; is about labelling and categorising; is retrospective, social and systemic and is about action and organising through communication. The process is iterative and is treated as a cycle rather than a linear sequence of actions. Key activities of sensemaking include noticing and bracketing, which happens when changes to flows of experiences occur and when people isolate the changes for closer attention to rationalise them [23]. Kolko terms this type of rationalisation ‘framing’ and described it as a point of view shaped over a long-term aggregation of thoughts and experiences [24]. Hence, the role of context, which involves factors or mechanisms influencing rationalisation, is important in the sensemaking process [25].

Using this perspective of sensemaking as our theoretical framework, we were not only concerned with what was explicitly stated as barriers and facilitators to adoption but why stakeholders decided in favour or against a solution and the rationale they used to derive at the decisions.

## Methods

### Rationale

This was a qualitative synthesis with an interpretive approach aiming to examine current knowledge by analysing previously published qualitative studies of stakeholder experience. The interpretive approach is usually inductive with the aim to develop a new concept or theory [26, 27]. In our interpretive approach, we drew on the framework synthesis method [28, 29]. In brief, this involved a systematic search for relevant publications, developing an initial coding framework based on the study aim, using an inductive approach to coding, whereby we developed new codes and categories in the framework as new factors were identified, interpreting the codes and categories for translation into themes, which were synthesised to aggregate findings, applying a sensemaking lens to further probe findings and to identify contexts within which SM solutions worked or did not.

### Data collection

We searched bibliographic databases and search engines: PubMed (Medline/NLM), Web of Science, LISTA (EBSCO), CINAHL, Embase and PsycINFO in July 2014 using a strategy aiming to capture studies reporting stakeholder experiences on the adoption or implementation of self-management interventions. Since self-management is an ambiguous term, the search strategy adopted multiple approaches using the following key search terms: self-management, chronic disease, adoption and implementation and key study design filters: ‘self-care’ and ‘self-monitoring’ to cross-check research results. We excluded systematic reviews and meta-analyses and intervention studies such as randomised controlled trials if they did not include any study of stakeholders’ experiences of the trial adoption, articles for which full text was not available, theory-based articles, and non-English language articles. We restricted our search to a five and a half year period (2009 to July 2014 considering this to be sufficient for the study aim similar to other qualitative syntheses [30].

An information specialist (NR) conducted the principal search with additional searching including forward citation and snowballing undertaken (JH).

**Fig. 1:**
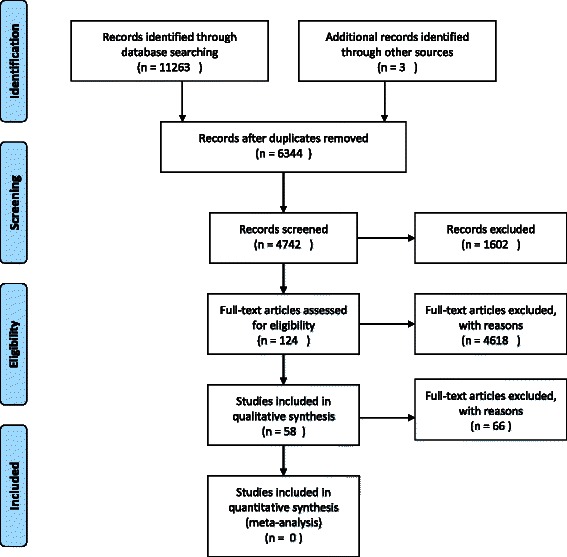
PRISMA flow diagram of search strategy used in the study

This strategy identified 11,266 publications. This number was reduced to 4742 after removal of duplicates, non-English articles, excluded studies and articles without full-text. The 4742 articles were filtered and screened for eligibility using the following search terms: experiences, implementation and adoption. From this, 124 articles were selected and screened individually to determine whether they were directly reporting stakeholder experiences of an intervention and 58 publications were selected to be included in our analysis [8, 9, 31–84]. Figure 1 shows our systematic search process.

### Data analysis and synthesis

The PDFs of included articles were downloaded into NVivo 10 and were coded and analysed inductively using the framework method described in Gale et al. [28]. In brief, this consisted of coding text from the publications as data into categories using Nvivo 10 Framework matrix and then developing interpretive concepts (themes) from the categories. JH conducted data extraction and coding. The coding framework was developed based on the study aim [85]; hence, we coded and analysed how self-management was described in the publications, barriers or facilitators to adoption, nature and context of the solutions, health conditions addressed, stakeholders and the conclusion points of the articles. Although new themes did not emerge after reviewing and coding three and a half years’ worth of publications (43 publications from 2011–2014 inclusive), coding and analysis continued to cover publication years 2009 and 2010 to ensure saturation was reached. The coding process and themes were reviewed by JP, and this included having access to, and checking the search strategy and results, the coding framework, resulting themes and the framework matrix. Each member of the team also had access to the framework matrix to check the codes and categories. Team members met to critically discuss results and the organisation of the paper.

Included articles reported 1300 semi-structured interviews (lasting from 30 to 90 min), 60 focus groups and 4700 survey responses. Other methods employed in the articles included ethnographic data analysis such as extended field notes from observations and shadowing, home visit field notes, chronic disease diaries, cultural probes, story sharing groups, case reflections, expert reflections, document analyses and enrolment logs with stakeholder comments. Data from these methods were collected from several perspectives including those of ‘patients’ such as expert patients, carers and patients’ families; ‘Health Care Professionals (HCPs)’ such as doctors, nurses, physiotherapists, medical assistants and psychotherapists and ‘managers’ such as programme (or trial) leaders, healthcare champions and policymakers (Table 1).

**Table 1:** Publications included in the synthesis

Journal title	First author: year of publication	Health condition	Country of study
From dictatorship to a reluctant democracy: stroke therapists talking about self-management.	Norris M: 2014	Stroke	UK
Implementing home blood glucose and blood pressure telemonitoring in primary care practices for patients with diabetes: lessons learned.	Koopman RJ: 2014	Type 2 diabetes	USA
Integrating a tailored e-health self-management application for chronic obstructive pulmonary disease patients into primary care: a pilot study.	Voncken-Brewster V 2014	COPD	Netherland
Barriers and facilitators to self-monitoring of blood glucose in people with type 2 diabetes using insulin: a qualitative study.	Ong WM: 2014	Type 2 diabetes	Malaysia
Faith wellness collaboration: a community-based approach to address Type II diabetes disparities in an African-American community.	Austin SA: 2014	Type 2 diabetes	USA
Why less may be more: a mixed methods study of the work and relatedness of ‘weak ties’ in supporting long-term condition self-management.	Rogers A: 2014	Chronic cardiac disease	England: UK
What matters to older people with assisted living needs? A phenomenological analysis of the use and non-use of telehealth and telecare.	Greenhalgh T: 2013	Multi-morbidity—old age	London and Manchester: UK
Barriers and facilitators to diabetes self-management: perspectives of older community dwellers and health professionals in China.	Shen H: 2013	Type 2 diabetes	China
Does telemonitoring in heart failure empower patients for self-care? A qualitative study.	Riley J: 2013	Heart failure	West London: UK
Home telehealth: facilitators, barriers, and impact of nurse support among high-risk dialysis patients.	Minatodani DE: 2013	End-stage renal disease	Hawaii
‘It is not going to change his life but it has picked him up’: a qualitative study of perspectives on long term oxygen therapy for people with chronic obstructive pulmonary disease.	Goldbart J: 2013	Long-term oxygen therapy	England: UK
Experiences of patients and professionals participating in the HITS home blood pressure telemonitoring trial: a qualitative study.	Hanley J: 2013	Blood pressure	Scotland: UK
2-year follow-up to STeP trial shows sustainability of structured self-monitoring of blood glucose utilization: results from the STeP practice logistics and usability survey (STeP PLUS).	Friedman K: 2013	Type 2 diabetes	USA
Is Europe putting theory into practice? A qualitative study of the level of self-management support in chronic care management approaches.	Elissen A: 2013	Various chronic conditions: cancer, cardiovascular disease, chronic respiratory illness, diabetes etc.	Austria, Denmark, England, Estonia, France, Germany, Hungary, Latvia, Lithuania, Italy, The Netherlands, Spain and Switzerland
Self-care in primary care: findings from a longitudinal comparison study.	Bagnall AM: 2013	Practice-based intervention	UK
GP support for self-care: the views of people experiencing long-term back pain.	MacKichan F: 2013	Long-term back pain	UK
Technology as system innovation: a key informant interview study of the application of the diffusion of innovation model to telecare.	Sugarhood P: 2013	Various conditions	UK
Internet-enabled pulmonary rehabilitation and diabetes education in group settings at home: a preliminary study of patient acceptability.	Burkow PM: 2013	COPD diabetes	Norway
Spanning boundaries into remote communities: an exploration of experiences with telehealth chronic disease self-management programs in rural Northern Ontario, Canada.	Guilcher SJ: 2013	Various: chronic lung disease, heart disease, stroke, arthritis	Canada
What happens when patients know more than their doctors? Experiences of health interactions after diabetes patient education: a qualitative patient-led study.	Snow R: 2013	Types 1 and 2 diabetes	UK
Exploring telemonitoring and self-management by patients with chronic obstructive pulmonary disease: a qualitative study embedded in a randomized controlled trial.	Fairbrother P: 2013	COPD	Scotland: UK
Care networking: a study of technical mediations in a home telecare service.	Correa G: 2013	Multi-morbidity—old age	Spain
Harnessing different motivational frames via mobile phones to promote daily physical activity and reduce sedentary behavior in aging adults.	King AC: 2013	Sedentary behaviour	USA
‘Getting the balance between encouragement and taking over’: reflections on using a new stroke self-management programme.	Jones F: 2013	Stroke	UK
Patients’ use of self-monitored readings for managing everyday life with COPD: a qualitative study.	Huniche L: 2013	COPD	Denmark
Ethical implications of home telecare for older people: a framework derived from a multisited participative study.	Mort M: 2013	Multi-morbid	England, Norway,The Netherlands, Spain
Patients’ experiences of shared decision making in primary care practices in the United Kingdom.	Fulwood C: 2013	Diabetes, COPD, IBS	UK
Chronic disease self-management and health literacy in four ethnic groups.	Shaw SJ: 2012	Various chronic conditions	USA
Remote participants’ experiences with a group-based stroke self-management program using videoconference technology.	Taylor DM: 2012	Stroke	Canada
Patients’ experiences of self-monitoring blood pressure and self-titration of medication: the TASMINH2 trial qualitative study.	Jones MI: 2012	Blood pressure, hypertension	UK
An organisational analysis of the implementation of telecare and telehealth: the whole systems demonstrator.	Hendy J: 2012	Multi-morbidities	Cornwall, Kent, Newhall: UK
Factors affecting acceptability and usability of technological approaches to diabetes self-management: a case study.	Vuong AM: 2012	Diabetes	USA
Diabetes connect: an evaluation of patient adoption and engagement in a web-based remote glucose monitoring program.	Jethwani K: 2012	Diabetes	USA
Supporting health behaviour change in chronic obstructive pulmonary disease with telephone health-mentoring: insights from a qualitative study.	Walter JA: 2012	COPD	
Mental health and relational self-management experiences of patients with type 2 diabetes and stage 3 chronic kidney disease.	Sakraida TJ: 2012	Type 2 diabetes and stage 3 chronic kidney disease	USA
Self-management experiences among men and women with type 2 diabetes mellitus: a qualitative analysis.	Mathew R: 2012	Type 2 diabetes	Toronto: Canada
Perceptions of effective self-care support for children and young people with long-term conditions.	Kirk S: 2012	Various chronic conditions	UK
One step at a time: self-management and transitions among women with ovarian cancer.	Schulman-Green D: 2012	Ovarian cancer	Connecticut, USA
Perspectives of patients with type 1 or insulin-treated type 2 diabetes on self-monitoring ofblood glucose: a qualitative study.	Hortensius J: 2012	Type 1 diabetes Type 2 diabetes	The Netherlands
Women’s experiences of factors that facilitate or inhibit gestational diabetes self-management.	Carolan M: 2012	Gestational diabetes	Australia
Social organization of self-management support of persons with diabetes: A health systems comparison.	Schiotz M: 2012	Diabetes	USA, Denmark
Exploring barriers to participation and adoption of telehealth and telecare within the Whole System Demonstrator trial: a qualitative study.	Sander C: 2012	Diabetes, COPD, heart failure, social care needs	Cornwall, Kent, Newham: UK
Self-care agency and perceived health among people using advanced medical technology at home.	Fex A: 2012	Diabetes, hypoxia, kidney disease, COPD, hypertension	Sweden
Factors affecting home care patients’ acceptance of a web-based interactive self-management technology.	Calvin KL: 2011	Chronic cardiac disease	USA
What motivates Australian health service users with chronic illness to engage in self-management behaviour?	Jowsey T: 2011	COPD, diabetes, chronic heart failure	Australia
Why do GPs hesitate to refer diabetes patients to a self-management education program: a qualitative study.	Sunaert P: 2011	Type 2 diabetes	Belgium
Integrating telecare for chronic disease management in the community: what needs to be done?	May CR: 2011	Various conditions including COPD	UK
Storylines of self-management: narratives of people with diabetes from a multiethnic inner city.	Greenhalgh T: 2011	Diabetes (not specified type)	UK
Self-monitoring technologies for type 2 diabetes and the prevention of cardiovascular complications: perspectives from end users.	Chudyk A: 2011	Type 2 diabetes	London, Ontario, Canada
Participants’ perceptions of the factors that influence diabetes self-management following a structured education (DAFNE) programme.	Murphy K: 2011	Type 1 diabetes	Ireland
Patient engagement with a diabetes self-management intervention.	Lidenmeyer A: 2010	Diabetes	UK
Determining clinical and psychological benefits and barriers with continuous glucose monitoring therapy.	Halford J: 2010	Diabetes type 1	Idaho Falls, USA
Technology enhanced practice for patients with chronic cardiac disease Home Implementation and Evaluation.	Brennan PF: 2010	Chronic cardiac disease	Milwaukee, Wisconson: USA
User acceptance of an Internet training aid for migraine self-management.	Sorbi MJ: 2010	Migraine	The Netherlands
Lessons learned from a collaborative to improve care for patients with diabetes in 17 community health centers, Massachusetts, 2006.	Lemay CA: 2010	Diabetes	Massachusetts: USA
Experiences of self-monitoring: successes and struggles during treatment for weight loss	Burke LE: 2009	Weight loss	USA
Barriers and facilitators to chronic pain self-management: a qualitative study of primary care patients with comorbid musculoskeletal pain and depression.	Bair MJ: 2009	Comorbid musculoskeletal pain and depression	Indianapolis: USA
Are some more equal than others? Social comparison in self-management skills training for long-term conditions.	Rogers A: 2009	Conditions receiving self-management education	UK

### Translating themes into aggregated findings

Themes were summarised into findings of what were identified as barriers and facilitators in the stakeholder groups. The studies identified many barriers and facilitators to adoption at individual and organisational levels most of which have been identified in other reviews [19, 86–92]. A deeper, more theoretical oriented analysis was then undertaken.

### Applying the sensemaking lens

A theoretical lens of sensemaking was used to analyse what were explicitly stated as barriers and facilitators in the studies and why they were put into these categories. To investigate the rationale behind stakeholder experiences as either facilitating or hindering, we analysed how adopters interpreted the solutions in relation to their daily lives by using author interpretation of the studies and direct quotes from participants in the articles. For context, we compared articles on similar solutions such as those for hypertension, diabetes, assistive technologies for multimorbid conditions and old age, stroke and self-management support. Using this analytical framework revealed that facilitators can also hinder adoption because adopters placed different values on what constituted a facilitator. In other words, one person’s facilitator may be another person’s barrier, and this appeared to be determined by how the solution was framed by the adopter in terms of value. Kolko describes framing as a point of view shaped over a long-term aggregation of thoughts and experiences [24].

In the following, we discuss results in the context of barriers and facilitators identified in the literatures from the perspectives of three stakeholder groups; we then discuss how adopters made sense of the solutions and how this appeared to influence uptake.

## Results and discussion

### Barriers and facilitators in the stakeholder groups

#### Patients

This group includes patients (including expert patients and user groups), their carers and families. In the literatures, patients used SM solutions to manage their conditions by engaging in activities such as setting goals, monitoring their condition and adjusting medication. From the patient perspective, factors influencing adoption of these solutions included knowledge of their condition [10, 11, 31, 35, 36, 38, 45, 50, 58, 62, 72, 82], their capability to comprehend and operate the solutions, their ability to embed or customise solutions into daily practices [33, 39, 41, 44, 46, 56, 57, 69], visible effect of the solution [10, 43, 66, 67, 74], cost and quality of the solutions and their effectiveness to manage the condition such as sending accurate readings [33, 35, 45, 52, 61, 70, 71, 79], family and healthcare professional support [9, 39, 40, 50–53, 58, 75], the need for motivational factors [35, 41, 45] and their ability to make decisions on adjusting medication independently [61, 79].

#### Healthcare professionals

This group consists of individuals and groups of clinicians such as doctors, nurses, physiotherapists, medical assistants and psychotherapists. In the literatures, HCPs used SM solutions to support patients’ management of their conditions such as reviewing the patient’s condition and feed back to them on their progress, providing support in goal setting and giving advice on medication dosage adjustment. In these tasks, HCPs, similar to patients, had to adopt a range of ‘technical’ and non-technical devices and processes into clinical practice. Factors influencing HCPs’ adoption included evidence that the solution works [14, 60], the solution’s alignment with goals of the organisation within which the HCP worked [37, 42, 68, 75], the integration of the solution into existing systems and practices [31, 32, 44, 60, 68], adaptability of the solution to learning and incorporating change [14, 33, 42, 51, 60, 68], transfer of decision-making power to patients and the effect of the solution on patient-doctor relationship [37, 49, 68, 71], time and resource constraints [54, 78], incentives and motivation to use the solution [14, 71], how the solution is promoted to the organisation within which the HCP worked [14, 51, 71, 75], HCPs’ appraisal of level of patient skill and interest in the solution [42, 71, 80] and adaptability of the solution to current roles and responsibilities [14, 32, 44, 49, 60, 68].

#### Managers

This group is made up of different levels of managers such as trial and intervention programme leaders, healthcare champions, policymakers and other managers with day-to-day responsibility of the trial or intervention. Murray and colleagues called this group implementers [15], and although we do not dispute this term, we will use ‘managers’ to describe this collection of stakeholders. In the literatures, managers were charged with responsibly delivering the solution to intended users on time and within budget and had tasks such as working with solution designers and developers, consulting with user groups, promoting the solution to users and documenting the effectiveness of the solutions. Along with Murray et al who described ‘implementers’ as a previously under-studied group [15], we also found that there is still very little literature on managers’ experiences. Factors affecting implementation included ability to deliver intended benefits of the solution, engaging effectively with business models, sustainable funding and resources, creating effective policies such as making adoption mandatory for HCPs, compatible commissions process across sectors and buy in senior leadership or active champions [14, 33, 47, 54, 78].

### How adopters made sense of the solutions

Adopters appeared to frame the solutions in relation to what the solutions *meant* to them in their daily goals and routines. This happened at both individual and organisation levels, as we will discuss in detail in the following sections. Therefore, what are traditionally conceived as facilitators such as hard and soft incentives (cost and added value); support from family, friends, colleagues or managers; desire to be seen as compliant (moral obligation); emotional motivation (psychological value) and the solution’s fit with existing practice and routines, could also be barriers, as discussed in the following sub-sections.

#### Cost and added value

In general, solutions are theorised to be more adoptable if they have cost-benefits and other added value such as socio-economic incentives, empowerment through gaining knowledge and expertise, power-sharing through collaborative decision-making, time and resource benefits and other forms of value [17, 19, 72]. Thus, our data showed that the cost of the solutions was a barrier for some adopters [43, 66, 79, 83], whilst in other circumstance buying equipment was less costly because it helped them monitor and managed their condition to prevent it from getting worse [31, 44, 57]. In a hypertension self-monitoring study for example, Hanley et al. [44] found that ‘*some were not concerned, did not think of their hypertension often and left the management to their doctor or nurse. For others, the diagnosis had caused practical problems (eg, in taking out life insurance)*’; hence, they purchased equipment to monitor and reduce risk, as these quotes from the study show [all quotes taken from the cited publication unless otherwise stated]:

I’m conscious of it because what I’m looking to do you do have to have a medical, and blood pressure is one of the key things that they don’t want, if you have high blood pressure you’re out. So I’m looking to get it down (Patient 20, monitoring group, no previous experience of home monitoring) [44].

I can’t remember if they…if I was advised to go and buy a home monitoring machine but I decided to do it anyway…I knew that my blood pressure would be checked every time, regularly at the surgery but certainly twice a year,… but until that I would like more information than that. (Patient 4, control group, previous experience of home monitoring with own monitor) [44].

In terms of other types of added value, patient empowerment through increased knowledge, independence and sharing decision-making has been depicted as a facilitator in the adoption of self-management interventions [4]; however, this was not always the case in the articles. A majority of the articles reported that patients felt empowered through increased knowledge of their conditions [36, 38, 65, 69]; however, some also reported that patients felt bound to their condition because of increased knowledge that reminded them of sickness, and so they would prefer to know less and enjoy life freely [35, 61, 65, 69, 79]. In this extract, for example, a participant explained why she did not always adhere to Self-Monitoring of Blood Glucose (SMBG):

December, I seldom check and I was away from the country also for a holiday. So, I let myself go during that time actually, don’t want to carry the strip, I mean the testing machine around to, you know to depress myself [participant giggled]. (P01, 57-year-old female clerk, diabetes for 17 years) [35].

Similar sentiments were evident in ‘shared-decision making’ in that whilst some individuals preferred to take active part in the decision-making on their conditions [37, 77], others were not confident or would rather leave decision-making to healthcare professionals, which decreased adoption [59, 79]. Hence, empowerment was a facilitator for some [10, 37, 39, 72, 77], but not for others [59, 72, 79]. In diabetes self-management for example, Murphy and colleagues found that ‘being in control’ was the overall outcome that could be expected when a person was empowered to implement the Dose Adjustment For Normal Eating (DAFNE) principles to self-manage their diabetes; however, some participants found that DAFNE took more time, and found the uncertainty unsettling and the self-responsibility difficult, and quoted a participant saying:

But I just have not got the determination nor the lifestyle, nor do I suppose really, do I want to be tied to it… [72].

Sugarhood and colleagues also found that some participants thought telecare reduced rather than facilitated independence:

Another risk mentioned frequently by participants was loss of independence. Use of a telecare device exposed the user to surveillance and control by social services [33].

Organisations also placed constructive and negative values on cost and the added value of self-management solutions. For example, patients’ ability to independently manage their conditions was generally depicted as facilitator, which could free up time and resources for HCPs, but when this facilitator did not align with organisational goals such as staying within prescribing budgets, it became a barrier. Snow and colleagues for example found that sometimes tensions rose between patient and HCP during the decision-sharing process if the patient made a request that increased short-term costs:

Using DAFNE principles, students learn how to achieve tighter, smoother blood glucose control by performing blood tests at least four times a day, using the results to make decisions about insulin dosage as well as ensuring that dangerously high or low blood sugars could be avoided. For some, this meant a considerable increase in test strip requirements as they took on the extra work to improve blood glucose control. Although course tutors provided letters for GPs explaining the necessity for these adjustments, interviewees reported having to be ‘quite tough’ to successfully negotiate with primary care teams who were reluctant to absorb the rise in short-term costs. Even when changes had apparently been agreed, they were sometimes reversed by healthcare staff without explanation or warning [37].

Hence, even if self-management facilitated organisational effectiveness in some ways, in other ways it appeared to be a barrier. Some HCPs were therefore not convinced of its relative advantage in terms of the costs and added value [33, 71].

#### Supporting use

Support is another factor widely identified as a facilitator in SM [58, 93]. In terms of organisations, HCPs expected interactive support from those implementing the solutions, such as training, manuals and workshops [42, 49, 54, 76, 78], but only attached constructive values, and were willing to adopt the solutions, if these types of support did not distract them from their duties. For example, in their study, which evaluated a self-care initiative at a practice level, Bagnall and colleagues found that one of the reasons why practices found it hard to assimilate necessary culture change needed to successfully implement the solution was the mismatched expectation of support and training by practice staff and noted that there were:

Problems in running the training packages as envisaged due to the level of commitment required from practice staff and mismatched expectations of the course content: Facilitators expressed difficulties gaining access to practices and arranging sessions. They found they had to shorten the programme and make it more appealing to practice staff. The feedback from the practices was that they expected to be told ‘how to’ implement self-care, whereas what they received were discussions on the nature of self-care. The package did in fact contain a number of ‘how to’ tools but interviewees did not mention these [54].

In terms of patients, the term ‘social support’ is an umbrella term used to describe supportive relationships built around the patient during adoption and can include the support of family, friends, peers and healthcare professionals [94]. Social support was strongly expressed in many of the publications as a facilitator to adoption [35, 58, 72–75, 77, 79, 81–83]. However, it also was reported that some patients felt ‘pressured’ from overly supportive family or friends, or felt they were being constantly reminded of sickness when with their peer group, and therefore experienced these types of support as a barrier [36, 62]. Some people, it was found, were motivated by social support such as enabling them to live longer and be near loved ones [36]; others however thought they were burdening family and friends, and since these patients did not want to become a burden, adoption was sometimes challenged. For example, in their investigation into motivational factors of self-management, Jowsey et al found that participants were motivated by support from family members but they sometimes expressed this in negative-sounding terms such as ‘family members *keep hassling*’ [36]. Similarly, a study on the mental health of patients who had type 2 diabetes and stage 3 chronic kidney disease also found that ‘*some family members, such as daughters and sons, were viewed as over-zealous in their effort to support diabetes SM, participants expressed a tension between pride of being cared about and a concern of being overly watched over*’ [62]. Furthermore, another study found that peer or group support was not always helpful as it depended on many factors such as social status, dynamics and how people legitimised their illnesses; in this study, it found that a young participant distanced herself from her group during self-management skill training because she did not feel her peers were like-minded:

the respondent who was young, 38 years old, had to give up working. Her main goal in life was to return to work and she had hoped that EPP would help her in this quest. In practice, she had difficulties, she felt the others in the group were too different so she got little from group affiliation or from attending the course [81].

#### Motivation

People attached different emotions to the solutions, and this either motivated or demotivated them. In some articles, it was reported that people associated self-management solutions with illness and stigma, old age, rejection by healthcare systems and inevitable death or boundedness (feeling bounded to the condition and its solutions) [46, 65, 69]. This decreased adoption. However, some attached completely opposite meanings. In the case of pendant alarms for older people for example, one study, from Spain, reported that the presence of technological agents made it possible for family members to remain present even in their absence; and although relatives were not at home, they were assured by the knowledge that they would be notified if needed and therefore the silent presence of the artefacts brings to the home the possibility for the constant presence of others [52]. In another publication on the implementation of similar technology in England, it was reported that whilst telecare was viewed ‘*as bringing an end to loneliness and isolation, some potential users assigned precisely the opposite meaning, linking the telecare device to social isolation and rejection by the healthcare system*’ [46]. Hence, telecare meant ‘the presence of family’ for some and ‘isolation and rejection’ for others.

#### Moral obligation

Some articles found that appealing to adopters’ desires to be good ‘citizens’ facilitated adoption and adherence [31, 35, 36, 46]. For example, Jowsey and colleagues found that the desire of patients to please clinicians and be seen as ‘good’ was found to be a motivator in adherence of self-management solutions [36]; Ong et al also found in diabetes self-management that ‘*participants claimed that they would “behave” and practice SMBG according to their physicians’ recommendations when their appointment dates were approaching. This is because participants wanted to show their physicians the “good” SMBG results so that their insulin dose could be decreased or they could have a longer interval before their next appointment*’ [35]. Whilst in another study, it was reported that patients felt that lack of support from and tensions in communication with clinicians, such as being chastised for abnormal readings, did not encourage them to be good in sustaining self-management [9]. Regarding HCPs, although offering extensive support to patients was generally depicted as a barrier due to lack of time and resources [11], Koopman and colleagues found that wanting to be a professional in providing a ‘good’ service to patients facilitated in adoption; therefore,

although the home monitoring data were electronically transmitted to the nurses, the nurses felt the need to continue a personal relationship with the patient, often by phone…Nurses felt obligated to touch base with patients, to give either instructions or feedback or just to let the patient know that they had reviewed the data [31].

Hence, people’s moral sense of ‘goodness’ can be a facilitator as well as a barrier in adoption.

#### Practices and cultures

In many of the articles, it was reported that aligning solutions with adopter’s existing practices and routines was a facilitator. Some however found this was not always facilitating because even solutions aligned to realistic situations required effort to learn and incorporate new skills that were not expected by the adopters. For example, it was contended that a key barrier to glucose monitoring in diabetes self-management was stress-causing procedures such as finger pricking; some of the studies went on to recommend other forms of monitoring that could address this barrier [35, 67, 74]. However, in a study that evaluated Continuous Glucose Monitoring (CGM), it was found that although patients’ were less stressed, 50 % of the study population stopped using CGM, and the second most cited reason for stopping was that the solution ‘did not meet expectation’ cited by 44 %. A conclusion from this study was that

Having patients identify if they are good CGM candidates should be part of the initial orientation process and occur before the decision to purchase a CGM system is made. Setting realistic expectations of what CGM can and cannot do will help increase patient satisfaction with the technology and reduce the incidence of non-use [79].

In terms of organisations, a major barrier discussed in the articles involved the solutions not being designed to align with practices such as their integration into existing organisational cultures and software systems to make them more accessible to staff [31, 32, 44, 60, 68, 75]. However, even where the solution had been tailored to imitate realistic practices, it was often found to be unsatisfactory, chiefly due to the time and effort required to learn the new solution. In one example, clinicians did not use data generated from blood glucose and blood pressure SM as they were not synthesised to reduce time and effort for interpretation [31]. Hence, factors such as learnability, readiness to adopt and expectations from the solution influence uptake in complex ways and cannot be simply classified as facilitators to adoption.

#### Facilitators changing to barriers during adoption

Sometimes constructive values attached to facilitative factors switched to the opposite during adoption. In the studies we reviewed, this switch from facilitator to barrier (or the other way round) appeared to be related to significant events such as (from a patient perspective) a death in the family, or watching family member develop complications, or (from the professional and organisational perspective) changes in the perception of risk or cost-benefit.

In one study for example, it was found that a patient who had monitored her weight and blood pressure twice a day via her telecare device experienced a ‘false reassurance’ and was very ‘upset’ when she suffered a heart attack [38]. Similarly, peoples’ past experiences with diabetes affected their decision to adhere to SM solutions. In one study, some participants became nihilistic having seen complications develop in family members leading to the view that diabetes was an ‘inevitably worsening condition’ leading to non-adherence to SM [75]; in other cases, participants saw positive effects on family members and therefore were keen to adhere [72, 74]. Hortensius et al. [67] found a similar friend and foe association in how patients with type 2 diabetes decided to adhere to self-monitoring of blood glucose, where patients were keen to continue using the intervention when they saw evidence of benefits such as positive visual effects of low readings but were hostile to using the solutions when evidence was not as expected. Other studies on blood pressure and chronic obstructive pulmonary disease (COPD) also found similar results in adherence [31, 43].

Similar to patients, values also changed for HCPs when something different was experienced or when their perception changed. Hendy et al. [68] for example observed that taking part in the intervention was initially perceived by the organisations as an exciting opportunity to obtain financial and management support (from Department of Health and a team of specialist management consultants) they needed to deploy telehealth on a large scale; however, once the intervention got underway, excitement was tempered by the level of work involved in developing new services.

### Discussion: implications for implementation

**Fig. 2:**
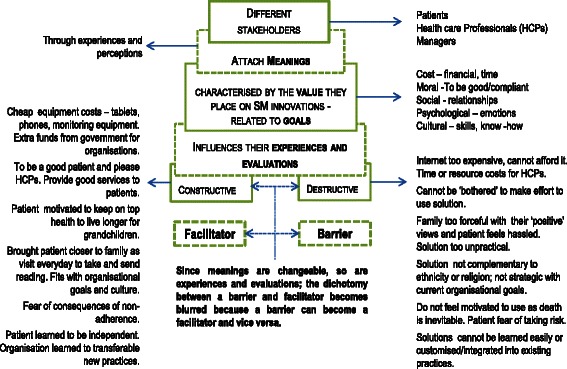
A graphical synthesis of the interchangeable nature of barriers and facilitators identified in this study

Overall, our analysis of the literature suggests that factors depicted as barriers and facilitators can have pluralistic (sometimes opposing) meanings, and these are influenced by the context within which stakeholders place SM solutions and of which stakeholders are apart. Taking into account that sensemaking is ‘an action oriented cycle that people continually and fairly automatically go through in order to integrate experiences into their understanding of the world around them’ [24], values related to cost, social, moral, psychology and culture played critical roles in how stakeholders made sense of the solutions; and this influenced their uptake and adoption. Citing Klein, Moon and Hoofman’s view [21], Kolko explains sensemaking as a process that is both personal and shared, one that takes place over a long period of time and one that is heavily dependent on a perspective or point of view [22]. Therefore, the values placed on the solutions are related to how adopters ‘framed’ [24] them or, in other words, their understanding of what the solution ‘meant’ to them. For instance, ‘family support’ could be seen as a positive value, perhaps with the *meaning* that ‘I can be near loved ones’ or as a negative value, with a *meaning* of (unwanted) dependency. Since sensemaking is a continuous process, these values are not static but dynamic, and meanings can change when a difference experience occurs or when a different notion is conceived (Fig. 2).

Reframing, or changing meanings, present a challenge for managers, who are charged with implementing, diffusing and sustaining solutions, sometimes, at scale. Managers implementing solutions are guided by a protocol of generalisable factors which may include a list of barriers and facilitators; however, as we have shown, these are contingent and changeable and can increase or decrease adoption. As a result, it was noted in some of the articles that managers formed the view that some adopters were more hostile to new solutions than others. For example, May and colleagues found that ‘implementers’ viewed HCPs as hostile to new solutions [14]. Hence, managers looked for ways to identify adopters that would be more accepting of the solutions. In the wider literature, some have offered answers to this challenge such as Roger’s diffusion of innovations model, which suggests ‘promoting’ the solution to early adopters, who will then promote it to others [95], or Greenhalgh’s concept of ‘Bricolage and Bricoleurs’, which recommends giving tools of the solution to adopters and allowing them to decide how best to use them [46].

Others have attempted to address this challenge by categorising the approaches people take to adopt self-management interventions [77, 82]. Lindenmeyer, for example, categorised patients’ adoption of diabetes management intervention as ‘information seekers’ and ‘programme browsers’ and suggested that ‘programme engagers’ were most likely to adopt and sustain the intervention [77]. Burke et al also used categories such as ‘well-disciplined’, ‘missing the connection’ and ‘diminished support’ to describe people’s sustainable adherence to weight loss interventions [82]. Furthermore, Murphy et al showed that overall people desired to be in control of their type 1 diabetes and would like to sustain self-management; however, adoption depended on five interrelated factors (knowledge, empowerment, support, relationship shift and motivation), and they showed that the extent to which one factor mattered was dependent on other factors being in place [72].

What we have shown is that the dichotomy between a barrier and facilitator is not distinct when implementing or adopting SM solutions. We have also shown that dualistic aspects to barriers and facilitators exist in some cases. For example, family support being both a motivator and a deterrent in the same context. Whilst this was not evident in the studies, it is likely that there are cases where a facilitator or a barrier changes to become neutral, rather than to become the opposite, and possibly, this less dramatic change is less likely to be reported in previous work. Also, whilst contexts which are directly linked to the condition were influential, it is possible that other contexts that are not linked to the condition were also influencing how adopters reframed the solutions. Overall, our findings show that adopting a SM solution or innovation is a dynamic process; therefore, approaches other than barriers and facilitators should be considered. In particular, it might be useful to iteratively re-evaluate each potential facilitator and barrier in relation to the adopter’s framing of the solution and contextual factors influencing the process, and whether this has changed since the last evaluation. This would be done throughout the implementation process to help adopters retain constructive evaluations of the solution.

### Limitations of the study

We followed PRISMA guidelines where applicable, including using a systematic and comprehensive search strategy. Nevertheless, despite searching electronic bibliographic databases and using forward searching and snowballing techniques, it is always possible that some papers may have been missed. The selection of studies and coding of data were undertaken by one researcher; we did not undertake double-coding although a second researcher did check the coding process and emergent themes. For an interpretative synthesis, we did not feel it was necessary to have two researchers selecting and coding every study. The whole team contributed to the interpretation of the coded data, but we acknowledge that a team approach to interpretation will still be influenced by the individual backgrounds of the team members. Another limitation is that we are analysing the data and interpretations provided by other authors in their published studies, and we did not have access to their primary datasets. It is possible that there may be other data that they did not describe in their published studies that may challenge our conclusions. As a qualitative literature review, our findings are descriptive and we hope to bring new insights to a challenging issue; it will be for future studies, and implementation projects, to test these insights in primary research.

## Conclusions and recommendations

This study critically challenges the notion of guiding implementation with preconceived barriers and facilitators, because every perceived barrier or facilitator has a possibility of having an opposing effect. We therefore suggest that it would be more helpful for those involved in implementation to consider factors that could help adopters attach constructive meanings when they frame and reframe the solutions in relation to their daily routines and goals. In practical terms, it is important to consider how to promote and sustain constructive meanings in relation to (a) cost: how much resource will the intervention cost the patient or professional; (b) moral: to what extent will people adhere because they want to be ‘good’ patients and professionals; (c) social: the expectations of patients and professionals regarding the interactive support they will receive; (d) motivational: motivations to engage with the intervention and (e) cultural: how patients and professionals learn and integrate new skills into their daily routines, practices and cultures.

Future studies can explore the contingent nature of barriers and facilitators and the importance of meaning. These should take the perspectives of multiple stakeholders (including patients, professionals and managers), with further exploration on what roles socio-cultural processes play in determining the values people place on SM solutions.
